# Feasibility and Acceptability of Utilizing a Smartphone Based Application to Monitor Outpatient Discharge Instruction Compliance in Cardiac Disease Patients around Discharge from Hospitalization

**DOI:** 10.1155/2014/415868

**Published:** 2014-12-11

**Authors:** Aimee M. Layton, James Whitworth, James Peacock, Matthew N. Bartels, Patricia A. Jellen, Byron M. Thomashow

**Affiliations:** ^1^Division of Pulmonary, Allergy and Critical Care Medicine, Department of Medicine, Columbia University Medical Center, VC3-365 Center for Chest Disease NYPH-CUMC, 622 W. 168th Street, New York, NY 10032, USA; ^2^Department of Biobehavioral Sciences, Teachers College, Columbia University, 522 W. 120th Street, New York, NY 10027, USA; ^3^Division of Cardiology, Department of Medicine, Columbia University Medical Center, 622 W. 168th Street, New York, NY 10032, USA; ^4^Department of Physical Medicine and Rehabilitation, Montefiore Medical Center, 111 E. 210th Street, Bronx, NY 10467, USA; ^5^Center for Chest Disease, New York Presbyterian Hospital, 622 W. 168th Street, New York, NY 10032, USA

## Abstract

The purpose of this study was to determine the feasibility and acceptability of utilizing a smartphone based application to monitor compliance in patients with cardiac disease around discharge. For 60 days after discharge, patients' medication compliance, physical activity, follow-up care, symptoms, and reading of education material were monitored daily with the application. 16 patients were enrolled in the study (12 males, 4 females, age 55 ± 18 years) during their hospital stay. Five participants were rehospitalized during the study and did not use the application once discharged. Seven participants completed 1–30 days and four patients completed >31 days. For those 11 patients, medication reminders were utilized 37% (1–30-day group) and 53% (>31-day group) of the time, education material was read 44% (1–30) and 53% (>31) of the time, and physical activity was reported 25% (1–30) and 42% (>31) of the time. Findings demonstrated that patients with stable health utilized the application, even if only minimally. Patients with decreased breath sounds by physical exam and who reported their health as fair to poor on the day of discharge were less likely to utilize the application. Acceptability of the application to report health status varied among the stable patients.

## 1. Introduction

In order to improve healthcare quality for patients with several chronic conditions, the Patient Protection and Affordable Care Act of 2010 instituted penalties for hospital reimbursement if a patient admitted for myocardial infarction, congestive heart failure, or pneumonia was readmitted to the institution within 30 days of the original discharge (Patient Protection Affordable Care Act 2010). As such, hospitals have been faced with identifying patients in these cohorts who may be at high risk for hospital readmission and implementing interventions in hopes of improving patient care and reducing penalties that may be incurred by early readmission [1, 2].

Multicomponent interventions that feature early assessment of discharge needs have been found to be beneficial in reducing readmission rates [3]. These multicomponent interventions have made use of education, timely transfer to primary care teams, post-acute followup between 24 and 72 hrs by nurse of physician, and appropriate referrals to support services such as rehabilitation programs [3] and the use of home exercise programs [4]. Although these programs may be effective, the extensive time and staffing needed for their implementation and success can limit their feasibility [5]. Alternatives to one-on-one patient to healthcare provider interaction have begun to be developed to potentially allow for a greater implementation of such interventions [6]. Various telemedicine approaches, such as smartphone applications, are being considered as potential tools for allowing healthcare professionals to implement and monitor patients remotely.

Smartphone applications are inexpensive and, unlike other forms of telemedicine, do not require home installation [7]. Smartphone applications can be utilized not just for accessing and tracking health information but also as a tool allowing practitioners and the patient's social support system to become more involved in his/her care without being physically present and in educational content delivery [8].

Areas where wireless health monitoring has been found to have succeeded in monitoring and tracking patients' health have been in patients with heart failure [9]. Patient's weight, blood pressure, and symptoms have been successfully monitored remotely by several telemedicine or structured telephone support systems [10]. However, these studies utilized multifaceted systems that provided and received information by more than just a smartphone application. Also the adoption of patients to utilize telemedicine systems has been an issue [11]. Chaudhry et al. found that recently discharged patients with heart failure had poor adherence of using the telemedicine system given to them after discharge. Given the age and demographics of heart failure patients, it is not clear whether a strictly smartphone application would be well adopted or demonstrate similar adoption problems as other telemedicine systems. Although there are potential benefits of utilizing smartphone technology for monitoring patients who are at high risk for being readmitted, the feasibility of monitoring heart failure patients or postmyocardial infarction patients via a strictly smartphone application has not yet been well documented. To our knowledge, there has been no report on the frequency of application use or potential barriers these patients may have in utilizing such technology after hospitalization.

The purpose of this study was to investigate the feasibility and acceptability of a smartphone iOS application to monitor and assist with patient medication compliance, education, home exercise, symptom changes, and transition to outpatient care team after hospitalization. The aim of this study was to determine the frequency of application use, potential barriers of use, and potential dropout rate in collecting this type of data in such population.

## 2. Methods

This study was a qualitative study discussing the home-based feasibility and acceptability of utilizing a smartphone application to collect health information and interact with patients with CHF or CAD around discharge.

The iOS application administered daily educational material, medication reminders, doctor appointment reminders, and monitored activity level. Process measurements, such as user engagement, daily task completion, and perceived value of the application to the patient, were recorded. Secondary outcome measures were activity level, medication compliance, follow-up care, enrollment into support programs such as cardiac or pulmonary rehabilitation program when applicable, and 30–60 day readmission rates in our population. Patients were given the usual standard of care throughout the study and the iOS application was only used to supplement outpatient discharge instructions and compliance, not to be used in place of the usual standard of care. Patients were instructed to follow all of their doctors' and nurses instructions and if there were any questions or confusion to contact their physician. All treating physicians were notified of their patient's participation in the study.

### 2.1. Participants

Hospitalized patients with a diagnosis of coronary artery disease (CAD) or congestive heart failure (CHF) were recruited. Patients were not considered for enrollment until clinical staff informed the study personnel that the patient would be eligible for discharge within the next 3 days. Study exclusion criteria consisted of a lack of described diagnosis, inability to perform physical activity, unstable angina, neurological deficit that makes the individual unable to understand and follow directions, being illiterate, non-English-speaking, and no home WiFi connection.

Physicians and nurses working on the inpatient cardiac units identified 180 patients for the study team who were admitted with a primary diagnosis of CAD or CHF and were eligible for discharge. The study team then approached the patients and screened them for patient interest and eligibility. The study staff made it clear that participation was completely voluntary and all usual care would continue regardless of study participation. If the patient demonstrated interest in participation, the study staff informed the patient of what study participation would involve and received his/her consent to participate. This study was approved by the Columbia University Medical Center's Institutional Review Board. All participants signed informed consent prior to participating.

### 2.2. Protocol

**Figure 1:**
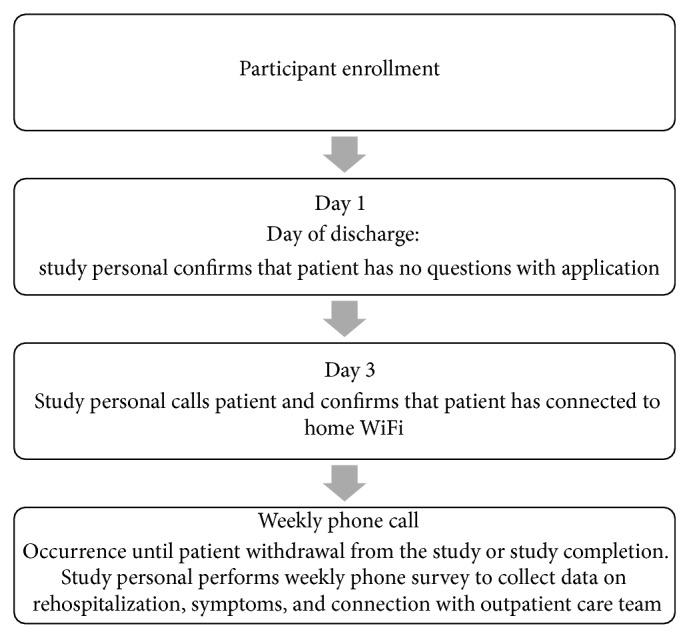
Study protocol.

Once patients consented to participating in the study, either the iOS application (Wellframe Application by Wellframe, Cambridge, MA) was uploaded to their smartphone or, if the patient did not own a device that could operate an iOS application, an iPod touch (iPod touch model A1367, Apple Inc., Cupertino, CA) was lent to the participant for the duration of the study. Patients were then given an orientation to how to use the device. A one-page instruction sheet was also given to the patient and the patient was instructed to practice using the application during their hospital stay. Study staff then followed up with the patient prior to discharge to ensure that there were no further questions regarding the use of the application.  Figure 1 demonstrates the study personnel follow-up phone call protocol to collect information on rehospitalization, symptoms, and connection with outpatient care team.

**Figure 2:**
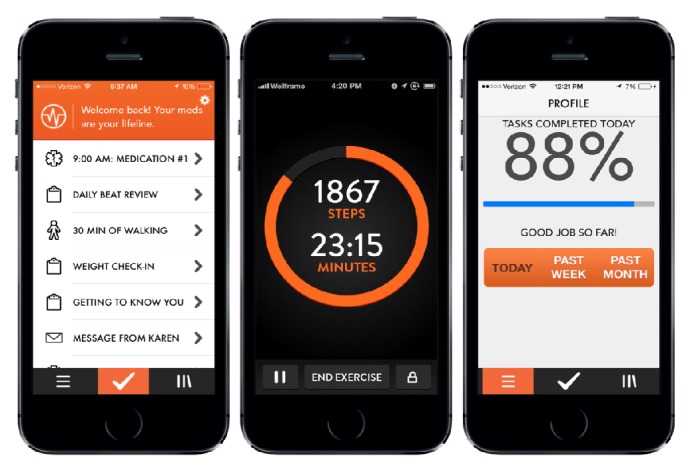
Example of application interface and content.

Each day the participants received reminders to take their medication at self-selected times, brief condition specific educational videos, and readings, and when relevant participants received reminders for upcoming appointments with healthcare providers. Additionally, participants could report changes in symptoms (e.g., dyspnea) and biometric measurements (e.g., heart rate) and track their physical activity through the iOS application's pedometer. Patients were considered “compliant” if greater than 50% of the “Daily To-Do Tasks” were completed.  Figure 2 depicts an example of the application's “Daily To-Do Tasks.”

### 2.3. Measurements

#### 2.3.1. iOS Application Content

The Wellframe application recorded medication compliance by self-report. The time of day the patient takes his or her medication was programmed into the application. Each day, at the programmed time, a medication reminder would appear on the patient's smart phone or iPod touch. The patient would then have to confirm by touching “yes” or “no” if he or she took their medication. If the patient selected “no,” an option to be reminded again in an hour would appear. If the patient selected “yes,” then a second reminder would appear on the phone or iPod touch an hour later.

Educational material was given to the patient daily via the patient's “Daily To-Do's.” The patient would be prompted to click on the educational reading material or video. Once the patient viewed the educational material, a check would appear on his or her “To-Do List” to demonstrate that task had been completed. The educational material consisted of disease management, smoking sensation, importance of attending cardiac or pulmonary rehab programs, and potential psychosocial issues associated with heart disease. Every day the patient received one of the “education topics” to read. Full list of topics can be seen in Table 1.

The patients were also sent daily messages encouraging him or her to perform daily walking, stretches, and light strengthening exercises (home based cardiac rehabilitation program) [12, 13].

When the patient tapped the prompt on his/her “To-Do” list to perform his/her daily exercises, he/she was then taken to a second screen that included instructions for the exercises, videos for the stretches and light strengthening exercises, and a pedometer for walking. At the end of the exercise regimen, the patient was asked to enter his/her level of breathlessness based on a modified Borg scale of perceived exertion [14]. For safety, the research team monitored the patient's exercises remotely and if a patient reported a level of breathlessness higher than 4 (somewhat severe), reported a heart rate exceeding 150 bpm, or had any adverse symptoms (e.g., dizziness, nausea, headache, chest discomfort, lightheadedness, drop in blood pressure, unusual heartbeat, or palpitations) the Medical Director of the Cardiac and Pulmonary Rehabilitation Program here at New York Presbyterian Hospital was alerted and a plan was in place to contact the patient. Patients were instructed to forgo the exercise program if they began a cardiac rehabilitation program.

Daily survey questions were asked regarding breathlessness, overall control of health, ease of using the application, medication side effects, and biometric measurements. Biometric measurements included weight, blood pressure, and heart rate.

During the weekly phone calls, the study staff would ask the patient if he or she had been enrolled in a cardiac rehabilitation program or had any upcoming doctor appointments. If the patient responded in the affirmative, then the staff would ask for the dates and times and enter the appointments into the patient's application via the dashboard. Staff also surveyed the patient to if he or she found the application useful and how is it most helpful. Lastly, the staff would inquire if the patient has seen his/her physician during the past week and if he or she has been recently hospitalized (if yes, then why). If the patient could not be reached after three attempts (over the course of 3 days), then a person the patient had designated as an acceptable emergency contact person for the study was contacted to confirm the patient's safety and if he or she had been hospitalized.

#### 2.3.2. The Dashboard

Patient data was uploaded to a remote dashboard for the study staff to view and send messages to the patient via a secure server (approved by Columbia University Information Technology Department as a HIPPA compliant and secure server approval forms attached). Data was uploaded real time and the study staff would contact the patient via the application 1-2 times per week to provide encouragement. The study team could also request a response from a patient via the messaging system on the dashboard and modify a patient's medication reminders or exercise protocol if necessary via the dashboard or by contacting Wellframe.

#### 2.3.3. Chart Review

Demographics, anthropometry, and ejection fraction and information about the patient's hospital stay were obtained by reviewing the electronic hospital chart. Information about any complications during the patient's stay, breath sounds by physical exam, and medications were retrieved from the physicians and/or nursing notes. For “day of discharge” information, notes within 24 hrs of discharged were considered acceptable and data from that note were retrieved. The pain scale and resting dyspnea scores were collected from the physical therapist's note on the day of discharge or the day prior to discharge if there was no physical therapy note provided at the day of discharge. Sleep quality and self-report description of healthy values were obtained via the Wellframe application at the day of discharge. All patients were asked to respond to the “daily beat questions” on the day of discharge so that the study team could ensure that the application was working properly.

### 2.4. Data Analysis

We tested the feasibility of using this application in patients with CHF or CAD after hospitalization by collecting data on the frequency of application use, usage barriers described by the patient population, study dropout rate, and type of data patients were more or less likely to provide.

Based on previous behavior usage research, investigating the usage of smartphone applications [15], we selected to analyze standard ethnographic and user data. Application usage was described by analyzing the frequency of response to symptom and biometric data survey questions, medication reminders, clicks on educational content, pedometer readings, and clicks of stretches and strengthening exercise videos. The logging in of system usage to detect adherence has been utilized in other telemedicine studies [11, 16, 17]. Study population size is similar to previous feasibility studies [17, 18]. Acceptability was tested by collecting data on application usage and types of questions patients choose to respond to about their health. To better define the type of patients that may be more or less likely to utilize the application, objective health parameters and self-described health status were collected. Patient's responses and usage were graphed to determine behavioral trends. Statistical analysis was performed using SPSS version 21 (IBM Corp., Armonk, NY, 10504). Nonnumeric variables (insurance, dyspnea, breath sounds, and description of health) were coded by severity. A linear regression test was used to determine whether any of the variables collected had a relationship with the amount with which a patient utilized the application. Significance was set a priori at *P* < 0.05.

## 3. Results

### 3.1. Participant Characteristics

**Figure 3:**
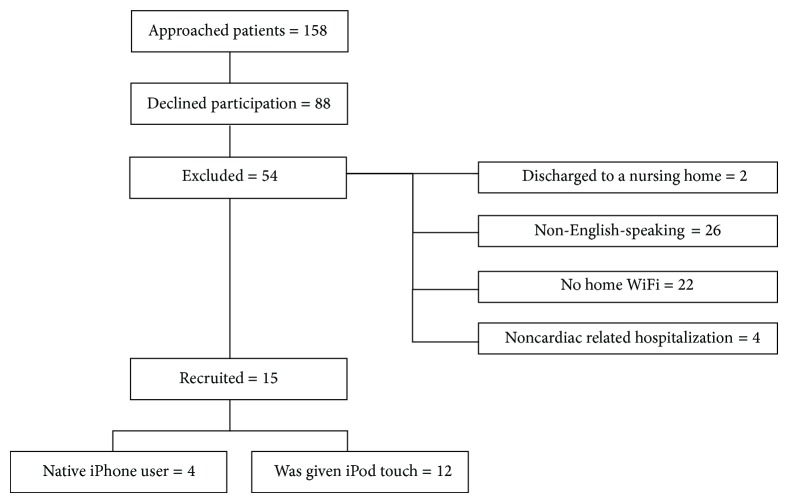
Participant recruitment and study population diagram.

Between July 2013 and December 2013, 158 patients were approached for the study. Twenty-two patients who were identified as eligible for the study were not approached because they were asleep or with a treating physician when study personnel attempted recruitment. In all of these occurrences the study personnel attempted to return to the patient's room for recruitment; however, the patient had been discharged.  Figure 3 depicts the breakdown of patients approached, declined participation, met exclusion criteria, and included. Of the 158 patients approached, 16 were enrolled in the study (12 M, 4 F, age 55 ± 18 years). Of the 16 patients, two were African American, one was Asian, one was Hispanic, and twelve were Caucasian. The largest barriers to enrollment were language and access to home WiFi.

Table 2 describes each patient's demographics, diagnosis, and socioeconomic status based on insurance provider. Ten of the patients who participated in the study had a diagnosis of CAD and were hospitalized for a cardiac intervention. Five of the patients were hospitalized with a primary admitting diagnosis of CHF. Most of our patients had private insurance providers.

### 3.2. Acceptability Analysis

The number of days patients interacted with the application after discharge and the characteristics of the patient's health are described in Table 3. The five patients who were readmitted to the hospital during the study duration (average of 7 days after discharge) did not utilize the application once discharged from the hospital. Of the remaining patients, all who were not readmitted, the application was utilized between 1 day and the complete 60 days. The average amount of days with which a patient utilized the application was 17. The average body mass index revealed that our patients were on average obese. The reported estimated ejection fractions by ECHO (EF) (taken after intervention when applicable) revealed that the majority of our patients had an EF of >55% except for 4 patients who had EFs of <45%. Of note, the patients who did not interact with the application were also the patients with more severe complications and complaints during their hospital stay, with the exception of one. This may reflect that sicker patients were less likely to utilize the application.

During the study, five patients (31%) were readmitted to the hospital, with the average readmission time of 7 days. Of note, all five patients only interacted with the application while being in the hospital and did not interact with the application once discharged. Of the 11 other patients, who were not readmitted during the course of the study, 8 interacted with the application within the first day after discharge, 1 interacted with the application 3 days after discharge, and 2 interacted with the application greater than 1 week after discharge (Table 1). Ten patients withdrew from the study prior to day 60; however, of these ten patients, five agreed to participate in the 60-day follow-up survey to verify if he or she had been hospitalized within 60 days after discharge. Four of the ten patients declined any further participation in the study. Retrospective chart review confirmed that all four patients were not readmitted to the hospital within 60 days of discharge.

#### 3.2.1. Correlates of Application Use

Results demonstrated that patients with unstable health after discharge had a lack of application use. All patients who were readmitted during the study did not utilize the application once discharged. These results are not suggesting a causational relationship but a correlative relationship, where application use may be a good indicator of overall health status.

Other correlatives of application use were breath sounds by physical exam within 24 hrs of discharge and the patient's self-report of health status at the day of discharge (Table 4). Analysis revealed that decreased breath sounds or crackles by physical exam within 24 hrs of discharge had a significant relationship with application use (*R* ^2^ = 0.370, *P* = 0.021). The patient's self-report of his or her health status at the day of discharge also had a significant relationship with application use (*R* ^2^ = 0.335, *P* = 0.038). Breath sounds and self-report of health were independent predictors of application use and could explain 79% of the variability in application use when modeled together (*R* ^2^ = 0.625, *P* = 0.02).

#### 3.2.2. Usage Barriers

The patients that found the application helpful also utilized the application the most and remained in the study the longest. Reasons patients gave to why the application was not helpful were the inability to change medication reminder times, the inability to enter doctor's appointments or other reminders themselves (the study team would have to enter these reminders into the dashboard and then the patient would receive the reminder), the pedometer would stop counting if another application on the device was opened (this is a limitation of iOS software design), and the general inconvenience of being asked to use the application on a regular basis. Upgrades to the application software and user interface have resolved many of these issues. Unfortunately, the inconvenience of entering data for a research study is a common limitation to participant compliance. Positive responses to application use were the usefulness of the medication reminders, the educational information provided in the “Daily Beat,” and the stretching and exercise videos.

### 3.3. Feasibility Analysis

**Figure 4:**
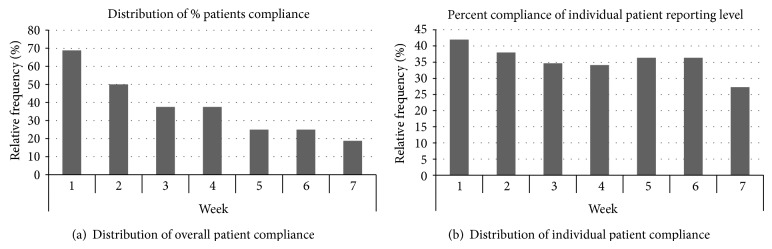
Distribution of compliance frequency.

Seven patients completed 1–30 days of the trial.  Figure 4 demonstrates the overall compliance of application usage. Sixty-nine percent of the patient population utilized the application during week one; however, the percent of relative frequency of use diminished to only 19% by week 7, demonstrating poor adherence over time (Figure 4(a)). During week 1, the patients answered the application questions with a median of 42% of the time. Over time, compliance diminished to a nadir of 27% (Figure 4(b)). On average, those patients utilized the medication reminders 37% of the time, read education material 44% of the time, answered survey questions through the application 55% of the time, and performed the recommended physical activity 25% of the time. Four patients completed 31–60 days of the trial and utilized medication reminders 53% of the time, read education material 53% of the time, answered survey questions through the application 93% of the time, and performed the recommended physical activity 42% of the time. The data reveals that the patients who completed 31–60 days of the trial utilized the application more than those who withdrew prior to day 31. Patients who answered most of the survey questions continued to answer most of the survey questions throughout the study protocol. Patients who answered fewer than half of the survey questions in the first week proceeded to drop out to the study the following week. A common theme of patients who completed greater than 31 days was the presence of a family member during study recruitment. It appeared that when the patient's family support valued the patient utilizing the application, the patient was more likely to utilize the application for >31 days and be more compliant with the use of the application.

Both groups participated with the survey tool that requested the patient to rate his or her breathlessness, take his or her pulse, enter his or her weight, describe his or her health that day, and respond to the ease of using the application's feature (Table 4). The patients who remained in the study the longest were the most compliant in answering these survey questions. Patients were more likely to answer the questions about shortness of breath, entering their weight and describing their health, and least likely to answer the questions regarding the ease of using the application or how in control of their health they felt they were (Table 4). In the patients who withdrew prior to day 31, they utilized the educational material the most and the physical activity aspect of the application the least. The patients who completed 31–60 days of the trial utilized the medication reminders and educational material equally.

## 4. Discussion

### 4.1. Principal Findings

This study has shown that collecting information regarding postdischarge compliance and patients' health status via smartphone application in patients with heart failure or coronary artery disease may be feasible but not without limitation. Our results indicate that patients who were medically stable were more likely to utilize the application than patients who are unstable. Results also demonstrated that patients were more likely to respond to medication compliance questions, read education content, and respond to a few survey questions but not all and were less likely to report physical activity through the application (Table 5).

Acceptability of the application was low but similar to that of other studies. Min et al. [18] reported a smart phone application median compliance rating of 41% in their population of women undergoing treatment for breast cancer. Chaudhry et al. [11] reported a 90% adherence in telemonitoring use in a population of patients with heart failure during the first week of the study but adherence decreased to ~55% by week 26. The low application compliance reported by this and other studies may reflect that although remote monitoring is convenient, the sample sizes needed for such research may be larger than those of other forms of survey tools or interventions. Therefore, future research in the field of remote monitoring may want to consider this low compliance rate when calculating desired sample sizes.

Compliance results also revealed higher acceptability rate in patients who remained in the study for greater than 31 days versus those who withdrew prior to 31 days. This relationship revealed a subset of patients who were more likely to utilize smart phone applications than others. Application usage is difficult to predict; however, work by Shin et al. [19] found that cellular network, time of day, and previous app use are all highly related to application usage. Hospitals that are seeking to utilize this kind of technology to track and interact with high risk patients may want to take into account these factors to optimize usage. Companies seeking to design applications for this use may also want to take into account these factors when designing their application.

Factors that impacted acceptability or barriers for usage were reported to be the inability to interact with the application as much as the individual required. As the patient's health and needs evolved, the application needed to be able to evolve. Also in patients whose health status was deteriorating, the application usage was decreased. These findings demonstrated a utility with smartphone applications to identify patients that need a care team to intervene rather than relying on technological remote monitoring. As highlighted by Pandor et al. [10], remote monitoring of patient's with HF health does not appear to impact the course of the patient's health unless monitoring dictates an action. Even when the remote monitoring dictates an action, study results have not always been positive [11]. Telemonitoring did not appear to provide a benefit over usual care when used to decrease rehospitalization [11]. Therefore, remote monitoring appears to be most useful in highlighting changes in behavior, rather than eliciting changes in outcomes. Thus, data collected by remote monitoring may be most useful for hospitals that are looking to allocate resources towards the patients who are most likely to be readmitted, rather than replacing usual care [11]. These results are not suggesting a causational relationship but an associative relationship between health status and application use. It is important to stress that smartphone applications may be most successful in helping to bridge the communication between care team and patient rather than be used to replace the need for one-to-one in-person interaction.

A novelty of this application to the growing “telemedicine” market was the immediate feedback mechanism via the application to a dashboard the clinician can log into, rather than other immediate feedback systems such as phone or video conference. Additionally, the telemedicine systems that involve home setup can cause a delay in information being received by the hospital. The average readmission time in our patient population was 7 days and mode was 3 days, and thus there was value in the ability to follow patients' status upon immediate discharge. For the above reasons, more of the remote monitoring market is moving to application use. The results from this study can help guide researchers, administrators, and companies in what aspect of the application appeared to be valued by the patient, such as medication reminder and educational content, and what aspects were underutilized, such as the pedometer and ability to communicate with the study team via the application.

A barrier to utilizing certain telemedicine devices discussed in prior research has been the barrier of training staff and patients on how to use the technology appropriately and in a timely fashion [20]. Some physicians expressed frustration with the time and level of technological sophistication needed to utilize certain technology [20]. A benefit to this particular application was the intuitive and simple user interface that took almost no training. However, this application was very limited in the information the clinician can gain from it. Perhaps future versions or application will include interfacing with diagnostic equipment that can provide a pulse rate, distinguish an arrhythmia, and allow for photographs to be uploaded to the dashboard. This would expand the use of the application beyond distinguishing the patient's general health state. However the cost-benefit ratio must be considered. Gurné et al. [21] described the incremental costs of using some telemedicine systems as being greater than the benefit in some settings. With each interface that an application builds, the cost increases. Also the ethics of attempting to replace in-person interaction with a multidisciplinary team in high risk patient populations, such as the one studied, should be considered [21]. However, the use of such applications as a tool to extend the reach of the care team, rather than a replacement, may allow for an inexpensive and manageable technique to identify patients who are in need of such in-person care.

There were limitations to this pilot study. Our patient population was not representative of the average patient here at New York Presbyterian as our initial version of the application was only in English. Since the completion of the study a Spanish version has been developed. Further research is needed to determine if these findings can be extrapolated to the Spanish speaking population. The WiFi requirement also limited recruitment. It is our suggestion that future work should consider a data plan that allows the patient to utilize the device with or without WiFi, although this feature does add a further expense. Future work in a larger and more diverse population may be beneficial to confirm the relationships reported in these findings and allow for findings to be extrapolated to other patient populations. Lastly, the use of such application as a survey tool was useful however limited. A validated health questionnaire was not utilized for this study. This made the interpretation of the survey data limited. As a test of feasibility, the survey tool demonstrated that patients would respond to the daily questions and what questions the patients preferred; however, the applicability of these responses was limited.

## 5. Conclusion

This study demonstrated the feasibility and acceptability of utilizing an iOS application to monitor outpatient behavior in a group of patients considered to be at “high risk for readmission.” Findings demonstrated that patients with stable health utilized the application more than patients with unstable health. The acceptability of the application varied greatly. There remains a need to better define aspects of smart phone applications that will result in optimal patient compliance; however, these results demonstrated that usage alone may be a useful tool to highlight patients in need of closer monitoring.

## Figures and Tables

**Table 1:** Educational content topics.

Topic	General description of the following was included:
Coronary heart disease	Pathology, management, and symptoms

Medications	The value of medications, instructions for taking medications, and potential side-effects

Exercise	The benefits of exercise and guidance for exercising safely

Nutrition	General nutritional recommendations and benefits

Smoking	Benefits of smoking cessation/reduction and resources for quitting

Alcohol	General guidance and recommendations around alcohol consumption for cardiovascular health and recovery

Psychosocial	Information and resources around mood disorders

Biometric risk factors	Supporting patients to “know their numbers” (ie., blood pressure, weight/BMI, lipids, or oxygen when applicable)

**Table 2:** Demographics.

Subject	Age	Dx	Gender	Socioeconomic status by insurance provider
1	54	CAD	M	Private Ins
2	50	CHF	M	Medicare
3	84	CAD	M	Medicare
4	40	CHF	M	None
5	81	CHF	M	Medicare
6	49	CHF	F	Private Ins
7	37	CAD	F	Medicaid
8	40	CAD	M	Private Ins
9	69	CAD	M	Private Ins
10	60	CAD	M	Private Ins
11	26	CAD	F	Private Ins
12	72	CAD	F	Medicare
13	28	CHF	M	Medicaid
14	70	CAD	M	Medicare
15	71	CAD	M	Medicaid
16	46	CHF	M	Private Ins
**Avg**	**55**	**CAD** = **10**, **CHF** = **6**	**M** = **12**, **F** = **4**	**7** = **Private Ins**, **4** = **Medicare**, **3** = **Medicaid**, **1** = **none**
**SD**	**18**			

Dx: diagnosis, CAD: coronary artery disease, CHF: congestive heart failure, BMI: body mass index, Private Ins: private insurance company provider, EF: ejection fraction, M: male, and F: female.

**Table 3:** Days interacted with the application after discharge, anthropometric measurement, severity of heart disease measurements, and hospital stay information.

Subject	Number of days patient interacted with app after discharge	EF	BMI (kg/m^2^)	Length of hospital stay	Readmitted to hospital within 60 days		Complications or complaints post-op
1	0	65%	28.9	19	SOB	7 days	Afib, R diaphragm paralysis
2	0	20%	39.4	6	Infection	24 hrs	Day of discharge, complained of chills
3	0	65%	29.4	19	Thrombo-cytopenia	3 days	Volume overload, Afib, knee pain
4	0	60%	54.0	8	SOB chest pressure	3 days	SOB
5	0	10%	23.8	3	Cardiac arrest	~3 wks	None
6	1	63%	29.1	2	No		None
7	1	25%	44.6	12	No		None
8	1	55%	21.0	5	No		L foot pain
9	5	60%	26.4	5	No		None
10	13	55%	24.8	4	No		None
11	26	55%	21.3	5	No		Post-op anemia
12	30	60%	38.4	9	No		Back pain
13	40	55%	25.0	8	No		None
14	48	60%	27.1	9	No		None
15	52	55%	27.4	13	No		Post-op anemia
16	58	45%	24.1	13	No		None
**Mean**	**17 **	**51%**	**30.3**	**9**		**5** = **Yes**, **11** = **No**	
**Std**	**21**	**17%**	**9.2**	**5**			

App: application, SOB: shortness of breath, Afib: atrial fibrillation, R: right, L: left, post-op: postoperative, R/O: rule out, and std: standard deviation.

**Table 4:** Day of discharge information.

Subject	Pain scale	Resting HR	Dyspnea	BS on exam DOD	Day of discharge described health as	Discharged with pain medication
1	0	107	Moderate	Decreased, wheeze	Fair	Oxycodone, Tylenol
2	5	99	Mild	Decreased	Fair	Tylenol
3	0	74	Moderate	Decreased/crackles	Good	Oxycodone, Percocet
4	0	85	Very mild	N/A	Poor	None
5	0	93	None	Crackles	Good	Aspirin
6	N/A	96	None	Clear	Fair	Toradol, Tylenol
7	0	72	Mild	Decreased	N/A	None
8	5	95	None	Clear	N/A	Hydromorphone
9	0.5	71	None	Trace crackles	Good	Aspirin
10	2	88	None	Clear	N/A	Oxycodone, Tylenol
11	6	94	Moderate	Clear	Good	Tylenol w/Codeine
12	10	68	Moderate	Trace crackles	Fair	Aspirin, Vicodin
13	0	99	None	N/A	Excellent	Aspirin
14	4	86	Very mild	Clear	Good	Tylenol
15	0	100	Very mild	Clear	Good	Oxycodone
16	0	100	None	Clear	Very good	Tylenol, Morphine
**Mean**	**2**	**89**				
**Std**	**3**	**12**				

HR: heart rate, BS: breath sounds, DOD: day of discharge, std: standard deviation.

**Table 5:** Synopsis of survey questions and answers by patients after discharge.

Survey questions	Days after discharge						
	1–7 (*N* = 11)	8–14 (*N* = 8)	15–21 (*N* = 6)	22–28 (*N* = 6)	29–35 (*N* = 4)	36–42 (*N* = 4)	43–58 (*N* = 3)
How breathless do you feel?	54%	75%	100%	83%	100%	100%	100%
Weight (lbs)	50%	83%	40%	50%	100%	100%	100%
How easy do you find using this app?	22%	33%	Question no longer asked				
Please count your pulse for 20 seconds	31%	63%	83%	67%	100%	100%	100%
How would you describe your health today?	73%	33%	83%	100%	100%	100%	100%
I am in control of my own health	33%	67%	75%	75%	100%	100%	100%
Do you like the exercises on the app?	29%	67%	Question no longer asked				
